# *Jumping Translocation Breakpoint* Expression in Midgestation Mouse Embryos

**DOI:** 10.3390/ijms26209952

**Published:** 2025-10-13

**Authors:** Carley McGrath, Taniya M Jayaweera, Thomas Lufkin, Costel C. Darie, Anca-Narcisa Neagu, Petra Kraus

**Affiliations:** 1 Department of Biology, Clarkson University, Potsdam, NY 13699, USA; mcgratc@clarkson.edu (C.M.); tlufkin@clarkson.edu (T.L.); 2 Biochemistry & Proteomics Laboratories, Department of Chemistry & Biochemistry, Clarkson University, Potsdam, NY 13699, USA; jayawetm@clarkson.edu (T.M.J.); cdarie@clarkson.edu (C.C.D.); 3 Laboratory of Animal Histology, Faculty of Biology, “Alexandru Ioan Cuza” University of Iași, Carol I bvd. 20A, 700505 Iasi, Romania; aneagu@uaic.ro

**Keywords:** jumping translocations (JTs), *Jumping Translocation Breakpoint* (*JTB*), *Prostate Androgen-Regulated* (*PAR*), Epidermal Differentiation Complex (EDC), human 1q21, murine 3q, mouse embryo

## Abstract

Jumping translocations (JTs) can lead to partial trisomies. A breakpoint within the gene known as *Jumping Translocation Breakpoint* (*JTB*) has previously been associated with JTs involving the long arm of human chromosome 1 (1q). These 1q+ amplifications are frequently observed in cancer. *JTB* was initially mapped to the Epidermal Differentiation Complex (EDC) at 1q21, and earlier studies primarily focused on its role in malignant or adult tissues. Using updated genomic data, we refined the mapping of *JTB*. We employed RNA in situ hybridization (RISH) to visualize *Jtb* expression with organ, tissue, and cell-level resolution. We demonstrate that human *JTB* and murine *Jtb* are located outside the EDC. In midgestational wild-type mouse embryos, *Jtb* is expressed in multiple tissues, including the developing heart and vertebral column, and shows partial overlap with the expression of early markers of the neural crest cell lineage. Our findings suggest that the oncogenic potential associated with human *JTB* translocations is likely unrelated to its previously assumed location within the EDC.

## 1. Introduction

Jumping translocations (JTs) are a type of aberrant genomic rearrangement. Chromosome 1 JTs have been found to fuse with the telomeric repeats of recipient chromosomes, potentially resulting in partial trisomies of the 1q arm (1q+). They are frequently observed in myeloma and various other cancers, including breast and prostate cancer, as thoroughly reviewed elsewhere [1,2,3]. A breakpoint was identified between exons 4 and 5 of the gene *Jumping Translocation Breakpoint* (*JTB*), located on human chromosome 1 [3,4]. *JTB* encodes a highly conserved 146 amino acid transmembrane protein, comprising a signal peptide, an extracellular portion and a cytoplasmic domain [1]. JTs involving the *JTB* gene can result in the production of a truncated JTB protein that lacks the transmembrane domain, possibly impacting its biological function and contributing to pathological processes [3]. Genomic alterations involving the gain or amplification of 1q21 (1q21+) were frequently linked to the dysregulation of oncogenes and associated with different cancers [3,5,6]. JTB is evolutionarily conserved across diverse eukaryotic species (UniProt ID: O76095) [3,7,8]. A conserved gene structure of *JTB* orthologs has been identified in several primate species (UniProt ID: O76095) [1]. However, despite JTB being discovered over 25 years ago [3], very little is known about its biological function.

*JTB* has been identified as a transforming growth factor beta-1 (TGFβ1)-inducible gene [9]. TGFβ1 is a cytokine known to suppress tumor formation in some contexts and promote tumor progression in others [10]. JTB’s exact roles in neoplastic mechanisms still requires further investigations, as it can be found either upregulated or downregulated depending on the type of malignancy [11]. JTB is functionally associated with the chromosomal passenger proteins/complex (CPP/CPC), key regulators of mitosis [1,12]. *JTB*, also known as the prostate androgen-regulated (*PAR*) gene, was previously identified as a target of the androgen receptor (AR) signaling pathway, based on physiological and functional evidence such as dihydrotestosterone-induced expression changes in androgen-sensitive human prostate adenocarcinoma cells (LNCaP) and the restoration of androgen responsiveness following AR reintroduction in the human androgen-independent prostate cancer cell line (PC3) [13]. However, the study did not provide direct molecular evidence of a physical interaction between PAR and AR, such as ligand binding, receptor mimicry, or AR binding to the *PAR* gene. Rather than functioning as an AR itself, the findings suggested that PAR acts as a downstream target of AR signaling. Currently, there is no indication that PAR binds androgens, mimics receptor activity, or shares structural similarities with the AR protein.

The genomic proximity of *JTB* to the gene encoding cyclic AMP-responsive element-binding protein 3-like protein 4 (*CREB3L4*) [14], another AR-regulated gene involved in cell proliferation and endoplasmic reticulum (ER) stress response [15], supports the hypothesis that CREB3L4 and JTB may be co-regulated by AR and potentially contribute to tumor progression. This is particularly relevant in androgen-responsive cancers, such as prostate cancer and the luminal androgen receptor (LAR) subtype of triple-negative breast cancer (TNBC) [15]. Given their shared regulation by AR, both JTB and CREB3L4 may represent potential molecular targets or biomarkers in AR-driven malignancies. In prostate cancer, CREB3L4 plays a critical role in promoting cell proliferation and is functionally linked to AR-regulated oncogenic pathways [14]. It belongs to the old astrocyte specifically induced substance (OASIS) transcription factor family and has been shown to be upregulated by androgen stimulation in prostate cancer cells [16]. Emerging evidence suggests that CREB3L4 contributes to prostate cancer progression not only by enhancing cell proliferation, but also by influencing differentiation and survival pathways. These findings underscore the relevance of CREB3L4 as a potential biomarker or therapeutic target, particularly in hormone-resistant prostate cancer, where AR signaling remains active despite hormonal therapy.

In parallel, the Bcl2 apoptosis regulator (Bcl-2) and Bcl-2-associated X protein (Bax), which, respectively, inhibit and promote apoptosis, regulate programmed cell death through their relative expression levels [17]. The Bax/Bcl-2 ratio is therefore a key determinant of cell survival versus cell death. Downregulation of PAR expression increased the Bax/Bcl-2 ratio and Bax levels, thereby inducing G2/M phase arrest and apoptosis in PC3 prostate cancer cells. This supports PAR’s role in promoting the malignant phenotype of androgen-independent prostate cancer cells [2,12,13]. Consequently, JTB may also impact cell proliferation and cell cycle regulation, thereby contributing to tumorigenicity [1]. JTB is currently investigated as a potential biomarker in several cancer types, including hematologic malignancies, breast cancer and prostate cancer [1,2,8,13].

Based on previous mapping using sequence-tagged sites derived from a yeast artificial chromosome (YAC) clone mapped to human chromosome 1q21, *JTB* was initially placed within a ~2 Mb region known as the epidermal differentiation complex (EDC) on human chromosome 1q21.3–23.3 [3]. The human EDC contains a cluster of 62 genes belonging primarily to three major gene families involved in epidermal differentiation, contributing to the formation of the stratum corneum and enabling the skin’s barrier function [4,18]. Gene arrangements within the EDC are conserved and play important roles in the regulation of gene expression [19]. For example, loricrin (*LOR)* is a single coding exon EDC (SEDC) gene located within the complex, while members of the *S100A* gene family form the outer borders of the EDC [19]. Aberrant epigenetic modifications and altered expression of EDC genes, particularly *S100* genes, frequently contribute to various epithelial tumors [20,21]. Although YACs were valuable tools at the time, YAC chimerism could complicate genomic mapping. Advances in omics technologies have since improved physical maps and clarified synteny relationships between human and mouse chromosomes [22]. Here, we used updated genomic information provided by the National Center for Biotechnology Information (NCBI) and the National Institutes of Health (NIH) to determine the refined genomic location of *JTB* on human chromosome 1 and *Mus musculus Jtb* on mouse chromosome 3.

Developmental signaling pathways can be reactivated in cancer cells to promote their own survival, rapid proliferation, immune evasion, and cellular plasticity. Cancer cells can undergo an epithelial-to-mesenchymal transition (EMT), a process used by migratory cells during embryonic development, which enables cancer cells to metastasize. To date, JTB studies have largely focused on adult tissues, tumor samples and in vitro cell lines. Interestingly, Northern blot and proteomic analyses have shown JTB expression in multiple adult tissues, with upregulation sometimes observed in malignant compared to normal tissues [2,3,23,24]. The mouse embryo is a well-established model organism for studying early signaling events [25].

Based on updated genomic information, we designed probes and conducted RISH on sections of wild-type midgestation mouse embryos to complement earlier JTB Northern and dot blot studies of adult human tissues and cell lines [2,3]. Midgestation embryos (E11.5 to E13.5) were chosen because this developmental window provides a blueprint for all major organs while still involving significant embryonic growth, during which many tissues undergo further differentiation and maturation. We confirmed widespread *Jtb* expression observed in previous RNA blotting techniques, now with cellular resolution not previously reported, and mapped *Jtb* expression in the context of the EDC and migratory cell markers.

## 2. Results and Discussion

### 2.1. Revised Genomic Location of Human JTB and Murine Jtb in the Context of the EDC

**Figure 1:**
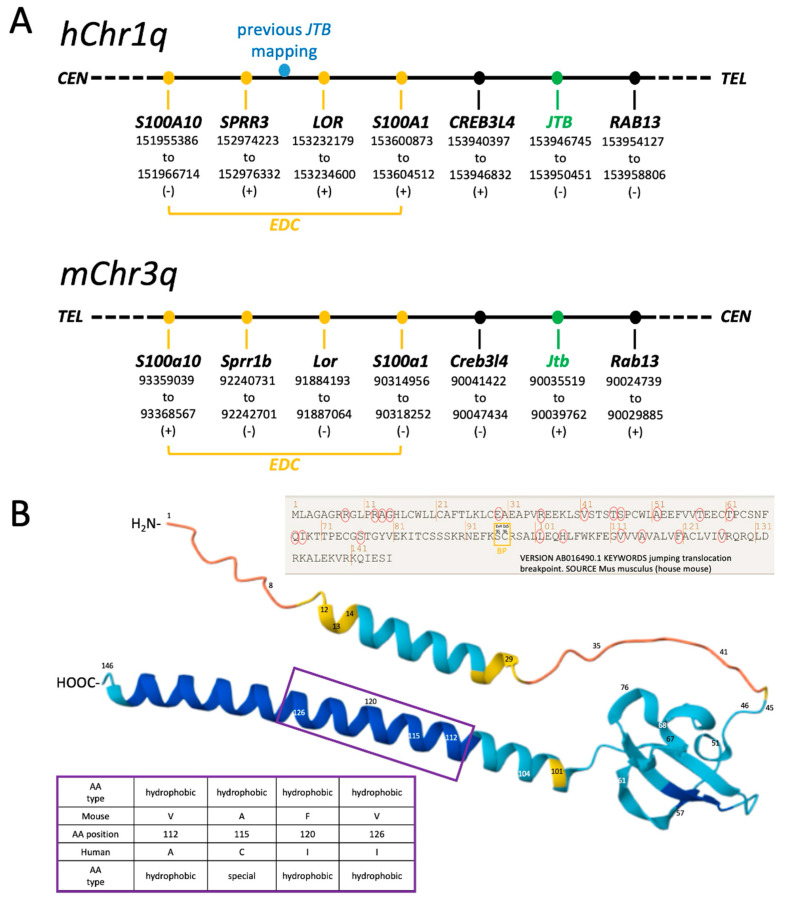
Location update. (**A**) Mapping of human *JTB* and murine *Jtb* based on NCBI data (not to scale). JTB/*Jtb* are located outside the EDC. (**B**) AlphaFold-based schematic showing differences in amino acid sequences between human JTB and murine Jtb proteins. Red circles refer to amino acid substitutions between mouse and human, the positions are indicated in the protein model. The purple box highlights several mostly conserved amino acid substitutions between mouse and human within the transmembrane domain.

The human *JTB* gene, also known as *PAR,* is located on human chromosome 1q21–23. Until now, *JTB* was reported to be situated within the EDC, and this EDC/*JTB* association was considered a potential contributor to tumorigenicity [3]. Our refined mapping, based on recent releases of the human (GRCh38.p14 (Build 37.2)) and murine (GRCm39 (Build 37.2)) reference genomes from NCBI, placed *JTB*/*Jtb* outside the EDC, which is delimited by *S100A* genes on both sides [3,19] (Figure 1A). Ideally, this revised mapping would be confirmed by super-resolution RNA fluorescence in situ hybridization (RNA-FISH) on human chromosome 1 and/or mouse chromosome 3; however, this is currently beyond our technical capabilities. The EDC plays a complex role in cell differentiation and epidermal development, requiring calcium-dependent protein cross-linking [19,21,26]. Genes within the EDC encode both structural and regulatory proteins crucial for the terminal differentiation of keratinocytes and for defining stratum corneum properties in mammals, reptiles, and birds [19]. *S100A* genes, which belong to the helix-loop-helix EF-hand motif gene family, encode proteins involved in calcium binding and facilitate crosslinking during the formation of the cornified envelope. They also participate in cell differentiation and cell cycle progression [19,27]. *Loricrin* (*LOR*, *Lor*), situated within the EDC, encodes a cornified envelope protein found in differentiated epidermal cells [3]. The new placement of *JTB*/*Jtb* outside the EDC requires a reassessment of the previously proposed correlation between the EDC, JTB, and cancer. We further used information from NCBI, combined with AlphaFold, to compare the predicted structures of human JTB and murine Jtb proteins. This analysis revealed several mostly conserved amino acid differences between the two species within the transmembrane domain (Figure 1B).

**Figure 2:**
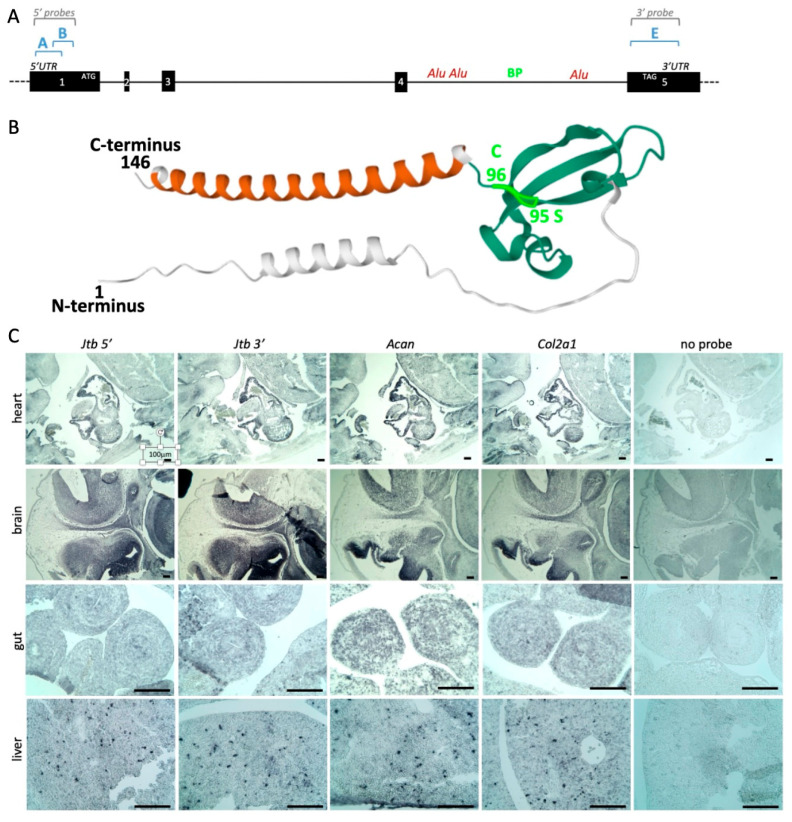
Murine *Jtb* expression in the context of extracellular matrix (ECM) markers. (**A**) Gene and probe locations drawn to scale. (**B**) AlphaFold model showing the location of the predicted breakpoint between serine (S95) and cysteine (C96). (**C**) *Jtb* probe A expression compared to that of ECM-related genes collagen type II alpha 1 (*Col2a1*) and aggrecan (*Acan*) in an E12.5 mouse embryo. Scale bar: 50 µm.

Advances in cancer and developmental biology have highlighted striking parallels between early embryonic development and tumorigenesis, including similarities in gene expression, proteomic profiles, and cell invasiveness [26]. Surprisingly, the role of *Jtb* in embryonic development has remained largely unexplored. Using the updated genomic data, we designed RISH probes to explore *Jtb* expression in embryonic tissues (Figure 2A, Table 1). Probes were designed to target regions upstream (5′) and downstream (3′) of the previously reported breakpoint located between *Alu* repeats in *intron4* [3]. We also used information from AB016490.1 (NCBI) and structural predictions from AlphaFold to align this breakpoint with amino acid 95 serine (encoded by *exon4)* and amino acid 96 cysteine (encoded by *exon5*) (Figure 2B). Additionally, we generated probes for EDC genes and for *Rab13*, a member of the RAS oncogene family. *RAB13* encodes a small GTPase involved in membrane trafficking and the regulation of epithelial apical junctions [28,29]. Despite multiple attempts, we were unable to define primers that would uniquely amplify *Creb3l4* for RISH probe generation. EDC genes, together with *CREB3L4* and *RAB13,* flank *JTB* on either side (Figure 1A). In humans, *RAB13* is located telomeric to JTB (towards the q-arm end of chromosome 1), while in mice, *Rab13* lies on the centromeric side of *Jtb* on acrocentric chromosome 3 (Figure 1). This positional difference could influence the outcome of a 1q+ jumping translocation, potentially leading to additional copies of RAS oncogene family member *RAB13* in humans, but not in mice.

In an initial RISH screen on sagittal sections of E12.5 wild-type mouse embryos, we compared *Jtb* expression with that of extracellular matrix (ECM) markers such as aggrecan (*Acan*) and collagen type II alpha 1 (*Col2a1*) (Figure 2C). The ECM provides structural support and organization to all tissues, including tumors, and plays a crucial role in tumor development and progression [30]. We observed overlapping *Jtb* expression with these ECM markers in several tissues, including the heart and gut, as well as punctate cellular expression in the liver. In the E12.5 brain, *Jtb* expression was more widespread compared to the ECM markers. Alkaline phosphatase (AP) reporter activity was not detected in the no-probe control sections (Figure 2C). Expression of the 5′ and 3′ *Jtb* probes appeared similar at E12.5. While serial sections were used whenever possible, it should be noted that this is a qualitative expression analysis, and differences in staining intensity between probes are more likely due to differences in probe length and A/T content than in transcript abundance.

### 2.2. Jtb Is Present in Many Crucial Tissues During Mouse Embryonic Development

**Figure 3:**
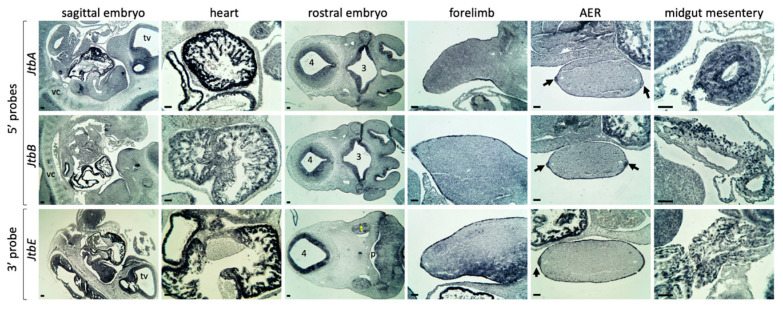
*Jtb* expression in E11.5 mouse embryos. Arrows indicate the AER (apical ectodermal ridge). Abbreviations: tv—telencephalic vesicle; t—trigeminal ganglion; p—branchial pouch; 3—third ventricle; 4—fourth ventricle; vc—vertebral column. Scale bar: 50 μm.

**Figure 4:**
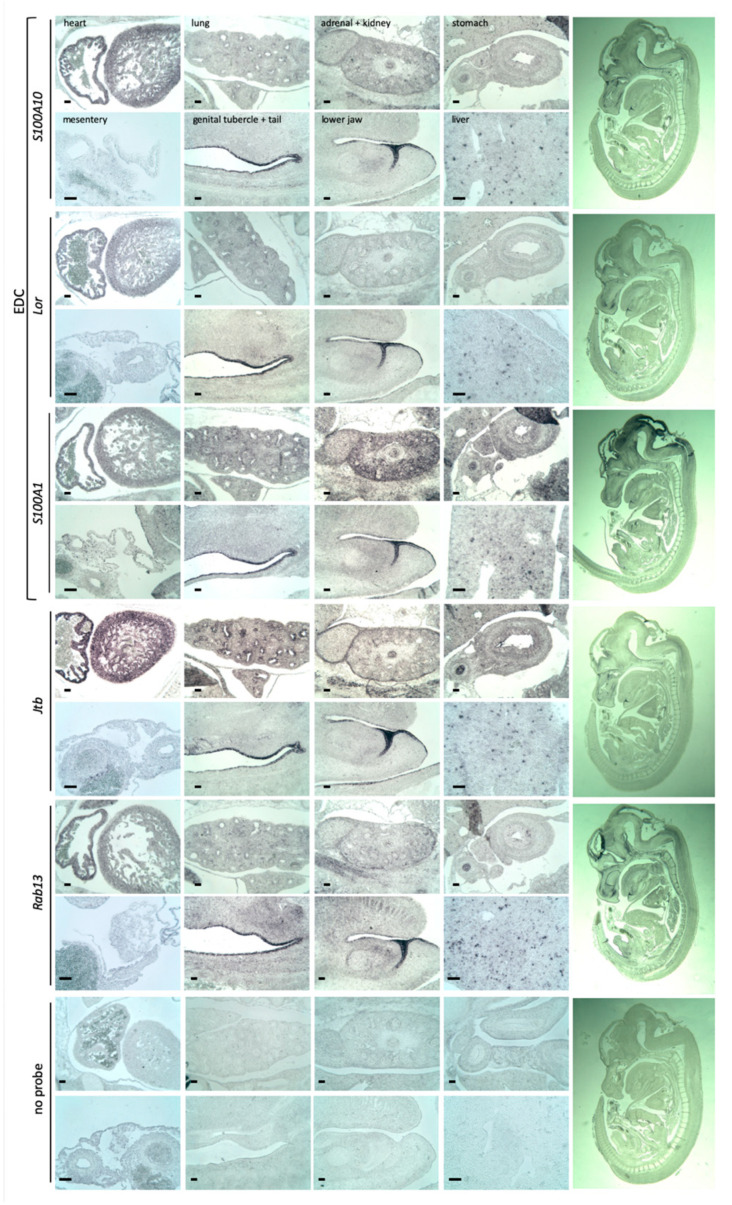
*Jtb* expression in relation to EDC genes and *Rab13*. RISH analysis on E13.5 mouse embryos using probes flanking *Jtb* on mouse chromosome 3 showed similar expression patterns. Abbreviations: EDC—epidermal differentiation complex; *Lor—*loricrin; *Jtb—*jumping translocation breakpoint, here, probe *JtbA*; *Rab13—*member of the RAS oncogene Rab family of small GTPases. Scale bar: 50 μm.

We extended our analysis to E11.5 wild-type embryos (Figure 3) and observed consistent *Jtb* expression in the heart wall and trabeculae carneae. Expression of *JTB* in heart tissue has previously been reported in adult samples by Northern blot [3]. We also noted *Jtb* expression in the developing nervous system, vertebral column and limb, particularly in the limb ectoderm and the apical ectodermal ridge (AER), a transient signaling center at this stage of embryonic development (indicated by arrows in Figure 3). In addition, we observed *Jtb* expression in cells of the developing lung, kidneys, and liver (Figure 3 and Figure 4). Expression was also seen in cross-sections of the midgut and in cells of the midgut mesentery at E11.5 (Figure 3).

The midgut mesentery, now considered an organ of mesodermal origin, is composed of connective tissue. Its associated cells include surface mesothelial cells, mesenchymal cells that give rise to connective tissue, and migrating enteric neural crest cells [31,32,33]. Our probe design enables detection of transcripts potentially affected by a *Jtb* jumping translocation (Figure 2 and Supplementary Material). However, the overall expression patterns of probes targeting upstream (5′) and downstream (3′) regions of the breakpoint in *intron 4* appeared qualitatively similar, with comparable spatial distribution in the tissues examined. This suggests the absence of a jumping translocation in a wild-type mouse embryo. It is important to note that this assay does not allow for quantitative comparison between probes. The apparently stronger signal from *JtbE* probe likely reflects differences in probe length and A/T content, rather than actual transcript abundance. Future quantitative analysis, such as RT-PCR, could provide further insights.

### 2.3. Jtb Neighbors

We compared the expression of *Jtb* with that of its chromosomal neighbors using serial sagittal sections of E13.5 wild-type embryos, including genes within the EDC, such as *S100a1*, *Lor* and *S100a10*, as well as *Rab13* (Figure 1A and Figure 4). All probes showed expression in the developing skin, as seen in the genital tubercle, tail region, and lower lip. While *Jtb* expression in cells of the midgut mesentery was less prominent than at E11.5, its expression in the heart remained strong. Among the EDC genes, expression in midgut mesentery cells was only evident for *S100a1*. This may be related to probe characteristics, although the probe length for *S100a1* was similar to that of *Lor*, for which no obvious expression was observed (Figure 4). In the bronchi of the lungs and in the esophagus, *Jtb* expression was again the most prominent, though expression of *S100* genes, *Lor,* and *Rab13* was also detected. In the developing kidney, *Jtb* expression was strongest in the medullary region of the metanephros and the surrounding connective tissue, while S*100a1* appeared most prominent in the kidney cortex. All probes showed punctuated expression in individual liver cells (Figure 4). Although overlaps in expression with *Jtb and* neighboring genes exist, *Jtb* also showed distinct expression features. RISH probes were specific for their targets, as confirmed by searches using the Basic Local Alignment Search Tool (BLAST) nucleotide suite (blastn) from NCBI (https://blast.ncbi.nlm.nih.gov/Blast.cgi?PAGE=Nucleotides, accessed on 30 September 2025) (Supplementary Material). Overlapping expression patterns may reflect broad regional epigenetic transcription control.

Also noteworthy is the previously mentioned genomic proximity between *JTB/Jtb* and *RAB13/Rab13*, particularly on human chromosome 1. Given the orientation of *JTB*, a jumping translocation involving its breakpoint in *intron 4* could lead to amplification of the RAS oncogene family member *RAB13*. This may represent an additional contributor to the reported association between *JTB* and malignancy.

### 2.4. Jtb Expression in the Context of Migratory Cell Markers

Given the broad tissue and organ distribution of *Jtb* transcripts during midgestation embryonic development, a potential association with multipotent migratory cells is conceivable. Such an affiliation could offer valuable insights into the observed association between *JTB* and various human tumor types.

Mesenchymal-to-epithelial transition (MET) is a fundamental process during embryonic development and organogenesis, including somite and heart formation. Its reverse, the epithelial-to-mesenchymal transition (EMT), also plays critical roles in development and tumorigenesis by promoting cell migration and enhancing the invasive potential of malignant cells [34,35]. Neural crest (NC) cells, multipotent progenitors with pleiotropic roles and contributions to multiple tissues, undergo both EMT and MET during development [36]. Here, we explored a potential overlap between *Jtb* expression and that of markers associated with migratory cells of the NC cell lineage [37].

**Figure 5:**
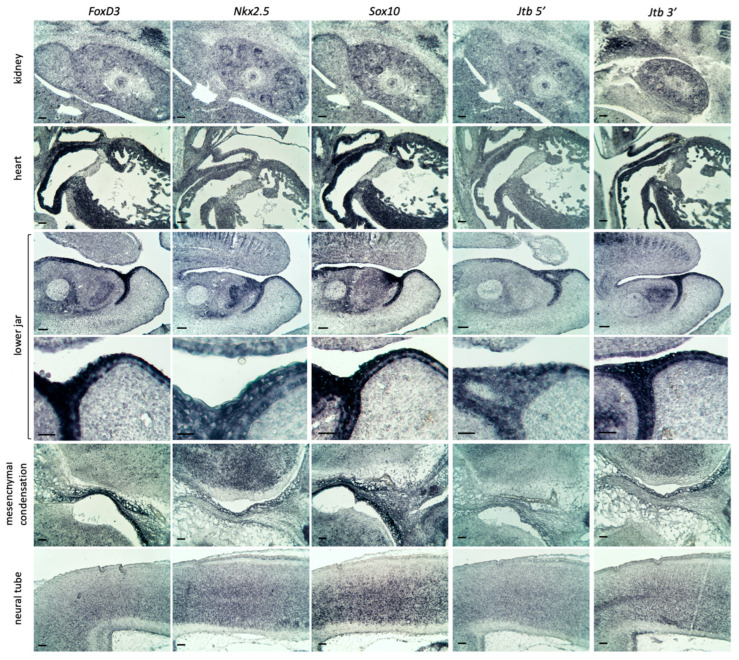
Expression of *Jtb* in the context of neural crest (NC) cell lineage markers (*Sox10, FoxD3*) and heart lineage marker (*Nkx2.5*) in E13.5 wild-type mouse embryos. Scale bar: 50 µm.

**Figure 6:**
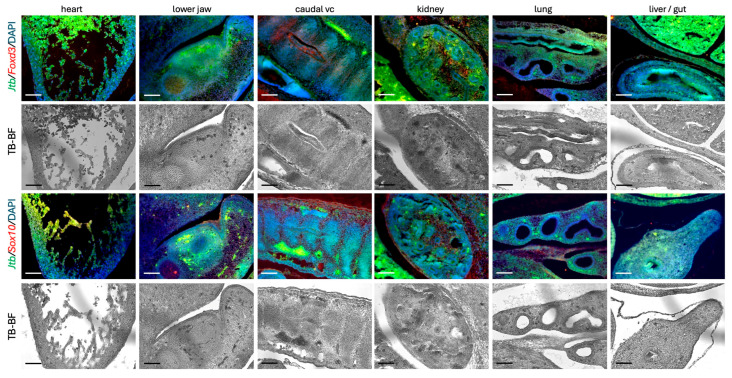
Double-RISH on E12.5 wild-type mouse embryos for the *JtbE* probe (Alexa Fluor 488, green) and neural crest cell markers, *Foxd3* or *Sox10* (Alexa Fluor 594, red). Co-expression ranged from white/yellow to light green in some, but not all, cells. Nuclei were counterstained with DAPI (blue). Corresponding bright-field (BF) images following TrueBlack^TM^ (TB) treatment are shown below each RISH image. Scale bar: 50 µm.

Two such NC lineage markers are the transcription factors Sox10, a SRY-box protein and NC tumor marker, and FoxD3, a forkhead-box protein and tumor suppressor. Both are key players within the NC gene regulatory network, controlling processes such as cell survival, migration, maintenance of pluripotency, EMT, and lineage commitment [37,38,39]. We also examined Nkx2.5, a homeobox-containing transcription factor (also known as Tinman in *Drosophila*), which is expressed in mesodermal and cardiac progenitor cells. While critical for heart development, it is not typically associated with the cardiac NC lineage [40,41]. In E13.5 wild-type embryos, we observed significant partial overlap between *Jtb* expression and these markers in several regions, including the heart, kidney, forming skin of the lower lip/jaw area, and neural tube. In the mesenchymal condensation and adjacent brain tissue in the embryonic head, the generally stronger *Jtb 3*′ probe signal most closely resembled the expression pattern of *Nkx2.5*. In contrast, NC markers were more strongly expressed in mesenchymal condensations, but less prominently in the adjacent neural tissue (Figure 5). Double RISH using fluorescent reporters suggested that *Jtb* is expressed in a subset of the NC-derived cells, as indicated by overlapping green and red fluorescence, producing white/yellow/lighter green signals in merged images, along with DAPI-stained nuclei (Figure 6). However, we also detected NC cells or derivatives without *Jtb* expression (red only), as well as *Jtb-*positive cells that did not co-express the NC lineage markers *Foxd3* and *Sox10* (green only) (Figure 6). Strong autofluorescence was noted in red blood cells despite quenching with True Black^TM^. Notably, alkaline phosphatase (AP)-based detection appeared more sensitive than fluorescently tagged probes, likely due to the accumulation of AP reaction product over time.

### 2.5. Jtb Is Expressed in the Developing Vertebral Column

**Figure 7:**
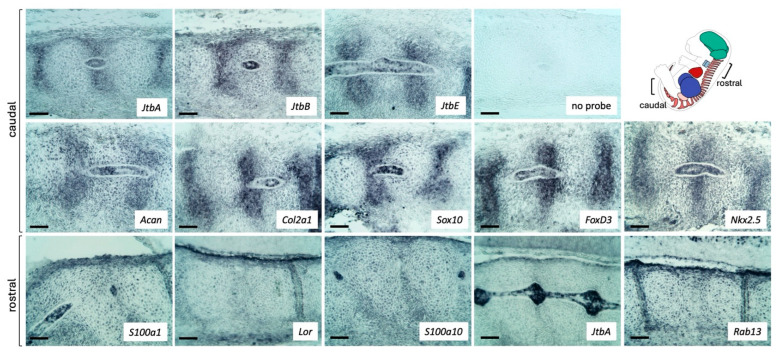
*Jtb* expression in the developing vertebral column of E13.5 mouse embryos. Scale bar: 50 µm.

Interestingly, we found *Jtb* expressed in the developing vertebral column, specifically in notochord-derived cells of the nucleus pulposus (NP), the central component of the future intervertebral disc (IVD). The notochord, like the AER, is a transient signaling center in the developing embryo (Figure 7) [42]. We observed *Jtb* expression overlapping with that of ECM markers, such as *Acan* and *Col2a1,* as well as with the early NC markers (Figure 7). This overlap is expected, as NC markers act upstream of ECM markers in signaling cascades that guide neural, glial, and chondrogenic cell differentiation [37]. Among the EDC genes, we found *S100a1* and *S100a10* also expressed in notochordal cells of the future NP, while loricrin expression was not notably detected.

## 3. Materials and Methods

### 3.1. JTB/Jtb Mapping Refinement

To update the *JTB/Jtb* locus within its chromosomal neighborhood, we used publicly available data provided by NCBI/NIH for both the human (GRCh38.p14 (Build 37.2), https://www.ncbi.nlm.nih.gov/datasets/gene/10899, accessed on 1 October 2025) and murine (GRCm39 (Build 37.2), https://www.ncbi.nlm.nih.gov/datasets/gene/23922, accessed on 1 October 2025) reference genomes and compared nucleotide positions for each gene. Advances in omics technologies have refined chromosome maps in recent years. After aligning *JTB* information as originally supported by STS and YAC data [3] and the EDC [4] with the genomic location updates by NCBI, *JTB* no longer resided between the *S100A* genes delimiting the EDC. While genomic NCBI/NIH data is publicly available, a *JTB/Jtb* location in the EDC remained in the recent literature and continues to be considered in the context of JTB’s involvement in malignancies.

### 3.2. Mouse Embryos

**Figure 8:**
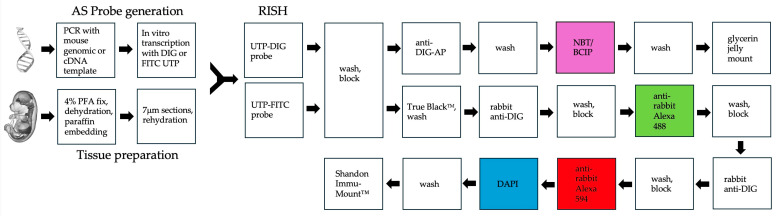
Workflow for chromogenic (AP) and fluorescent (Alexa Fluor 488/Alexa Fluor 594) RISH. While DIG- and FITC-labeled RNA antisense probes can be added simultaneously, primary and secondary antibodies must be added sequentially, as indicated. Abbreviations: AP—alkaline phosphatase; AS—antisense; PFA—paraformaldehyde; PCR—polymerase chain reaction; DIG—digoxigenin; FITC—fluorescein isothiocyanate; DAPI—4′,6-diamidino-2-phenylindole; NBT/BCIP—nitro blue tetrazolium/5-bromo-4-chloro-3-indolyl phosphate substrate; UTP—uridine triphosphate nucleotide.

Animal procedures were carried out following the Institutional Animal Care and Use Committee (IACUC) guidelines, as set by the National Advisory Committee for Laboratory Animal Research (NACLAR) under IACUC protocols No. 110689 and 110648. Embryonic day (E) 0.5 was defined as the morning on which a vaginal plug was observed in timed matings. Wild-type embryos of either C57BL6/J/129Sv background (Figure 2, Figure 4, Figure 5, and Figure 7) or CD1 background (Figure 3 and Figure 6) were harvested at E11.5–E13.5, fixed in freshly prepared 4% (*w*/*v*) paraformaldehyde (PFA), dehydrated, paraffin-embedded, and stored under dry conditions at room temperature until further use (Figure 8). Midgestation embryos E11.5–E13.5 were selected because all major organs were present at these stages. No obvious differences were noted between the strains.

### 3.3. RNA In Situ Hybridization (RISH) for Gene Expression Analysis

RISH was performed on sections of midgestational wild-type mouse embryos as previously described [43,44,45,46]. UTP-digoxigenin (DIG, Roche, Basel, Switzerland) or UTP-fluorescein isothiocyanate (FITC, Roche) labeled probes were generated through in vitro transcription from PCR-amplified murine genomic DNA or cDNA, using gene-specific primers. Reverse primers included a 5′ T3 or T7 promoter recognition site to enable the synthesis of antisense probes (Table 1, Table 2 and Table 3, Figure 8).

Alkaline phosphatase (AP)-conjugated anti-DIG antibodies (Roche), in combination with the nitro blue tetrazolium/5-bromo-4-chloro-3-indolyl phosphate (NBT/BCIP) substrate (Roche), enabled qualitative gene expression analysis on 7 μm sections of midgestation wild-type mouse embryos mounted on Histobond^+^ slides (VWR) (Figure 8, Table 1, Table 2 and Table 3). Slides were incubated for 24 h at 4 °C in the dark following NBT/BCIP substrate (Roche) addition, then fixed with PFA and mounted using glycerin jelly. Double RISH, including buffer compositions, was previously described in detail in Li et al. (2021) [47], and key steps are highlighted here (Figure 8 and Table 1, Table 2 and Table 3). The following primary antibodies were used for double RISH: rabbit anti-DIG (TFS) and rabbit anti-FITC (DFS). These were detected by secondary antibodies: goat anti-rabbit-Alexa Fluor 488 (TFS), representing *JtbE* transcripts, and goat anti-rabbit-Alexa Fluor 594 (TFS), reflecting *Foxd3* or *Sox10* expression (Table 1 and Table 3). TrueBlack^TM^ (Biotium) was used to reduce autofluorescence from red blood cells. Notably, fluorescence from the UTP-FITC was not detectable; red fluorescence originated from the Alexa Fluor 594 fluorophore. A flow chart outlining RISH and double RISH procedures is provided in Figure 8. Results were documented on a Motic BA310 compound microscope with a Moticam A16 16 MP camera (Carolina Biological) or a Zeiss Axio Vert for fluorescent images. RISH was performed at least twice for all probes.

## 4. Conclusions

Refined mapping based on available updated genomic data now places human *JTB* and murine *Jtb* outside the EDC. The provided *Jtb* expression data for midgestation mouse embryos indicated similarities between *Jtb* expression and its chromosomal neighbors, which could suggest a more global gene expression regulation in this chromosomal region. Epigenetic gene regulatory mechanisms implicating DNA demethylation, chromatin decondensation, and telomere dysfunction were previously suggested [48]. We confirmed the previously described expression of *Jtb* in the heart, but also showed its otherwise widespread yet not ubiquitous expression in midgestational wild-type embryos. Some similarity and even co-expression with NC markers was noted at this stage of development, suggesting *Jtb* could be expressed in cells of the NC lineage or their descendants. If *Jtb* is expressed in migratory cells of the NC cell lineage, this could provide one explanation for its association with many different malignancies [1]. However, based on the present data, we cannot conclude that *Jtb* is part of the NC signaling cascade. In vivo lineage analysis could further clarify this. To better understand the role of *JTB* in cancer, it will be necessary to carefully dissect breakpoint effects on JTB as a truncated protein and 1q21+ effects. A comparison of mouse and human translocation models would be helpful in the future.

## Figures and Tables

**Table 1:** Primers for RISH probes used in this study.

Probe	Forward Primer	Reverse Primer	Reference Sequence	Promoter	Product Length [bp]	Label/Fluorochrome for Double RISH
*Jtb A*	AGCCTCCGCGAGCAAGATG	GCGCTATAGGAACGGCCTCG	NM_206924.2	T7	174	
*Jtb B*	GACGCCTGCGTCCCACAA	AGCAGCCAGATGCCAGAGTTC	NM_206924.2	T7	130	
*Jtb E*	TGCCGTTCGGCTCTACTGGA	GCCGCTGAACTTTCTCCATCTGA	NM_206924.2	T7	222	DIG/Alexa Fluor 488
*Col2a1*	CCATTGCGAACCCAAAGGAC	CACCATTGTGTAGGACACGC	NM_031163.4	T3	326	
*Acan*	TGAGAGAGGCGAATGGAACG	CCAGTCCAGCCGAGAAATGA	NM_001361500.1	T3	998	
*S100a1*	TATGTTGTGCTGGTGGCTGCT	CCTTGGTGCACGTCGAGACT	AF368423.1	T7	288	
*S100a10*	CACCCACGGGGTCACTTGAG	TGAGGGCAATGGGATGCAAACA	NM_009112.2	T7	171	
*Lor*	TGGCAAGGGTGTGCCAGTCT	GGGGGAAGGGGCGCTTAAAAT	NM_008508.3	T7	291	
*Rab13*	TGGCACCTCAAGGGGAGATGA	GGCAAGGTTCCGTCCACTCT	NM_026677.4	T7	519	
*Sox10*	TCCAGCCAGGGTGTTTGGTG	CTCGTGAAGAGCCCAACGCC	NM_011437.1	T7	217	FITC/Alexa Fluor 594
*Nkx2.5*	GTGACGCAGAACTGCCCGT	TGGCGACGCAGGTTTCACAG	AF083133.1	T7	251	
*FoxD3*	AACTCAACCCGTCCGCTGG	ATAAAACTGCGCAGAGTGAACCTT	AF067421.2	T7	255	FITC/Alexa Fluor 594

**Table 2:** RISH protocols used in this study.

RISH-Type		AP-RISH	FL-Double-RISH	Comments
Steps	Repeats/Timing/Temperature			
Deparaffinization	3 × 20 min at RT	Histochoice		
Rehydration	each step 10 min at RT	ethanol gradient: 100%/100%/90%/70%/50%/30%/PBS		
Fixation		4% (*w*/*v*) PFA in PBS		
Wash	3 × 5 min at RT	PBS		
Prehybridization	2–3 h at 62 °C	prehybridization solution		prevent dehydration
Hybridization	o/n at 62 °C	prehybridization solution with DIG-labeled probe	prehybridization solution with DIG- and/or FITC labeled probe(s)	
Post-hybridizationwashes	3 × 20 min at 62 °C	solution I		
	3 × 5 min at RT	TNT		
	10 min at RT	TNT/solution II (1:1)		
	3 × 20 min at 58 °C	solution II		prevent dehydration
	3 × 5 min at RT	PBS		
Blocking	2 h at RT	SuperBlock^TM^ (PBS, TFS, Waltham, MA, USA)		
Primary antibody	o/n at 4 °C	anti-DIG-AP (1:2000)	rabbit anti-DIG (1:100)	
Wash	3 × 10 min at RT	PBS		
	6× hourly at RT	PBS	NA	
	3 × 10 min at RT	NTMT		
Color development	24–48 h at 4 °C in the dark	NBT/BCIP		
Blocking	2 h at RT	NA	SuperBlock^TM^ (PBS)	prevent dehydration
Secondary antibody	3 h at RT in the dark		goat anti-rabbit Alexa Fluor 488 (1:1000)	
Wash	3 × 10 min at RT away from light		PBS	
Blocking	2 h at RT away from light		SuperBlock^TM^ (PBS)	prevent dehydration
Primary antibody	3 h at 4 °C in the dark		rabbit anti-FITC (1:100)	
Wash	3 × 10 min at RT away from light		PBS	
Blocking	2 h at RT away from light		SuperBlock^TM^ (PBS)	prevent dehydration
Secondary antibody	o/n at 4 °C in the dark		goat anti-rabbit Alexa Fluor 594 (1:1000)	
Counterstaining	1 × 10 min at RT away from light		DAPI in PBS (1:1000)	
Wash	3 × 10 min at RT away from light	PBS		
Mounting	At RT away from light	glycerin jelly	Shandon Immuno Mount^TM^(TFS, Waltham, MA, USA)	
Imaging		Motic BA310	Zeiss Axiovert	

**Table 3:** Essential RISH chemicals/solutions used in this study.

Type	Chemical/Solution	Composition/Concentration	Supplier/ Order Number
Antibodies	anti-DIG-AP Fab fragments	1:2000 in SuperBlock^TM^ (PBS)	Roche #11093274910
	rabbit anti-DIG	1:100 in SuperBlock^TM^ (PBS)	TFS #9H27L19
	rabbit anti-FITC	1:100 in SuperBlock^TM^ (PBS)	TFS #71-1900
	goat anti-rabbit-Alexa Fluor 488	1:1000 in SuperBlock^TM^ (PBS)	TFS #A11008
	goat anti-rabbit-Alexa Fluor 594	1:1000 in SuperBlock^TM^ (PBS)	TFS #A11012
Solutions and stains	DAPI	1:1000 in water	TFS #62248
	DEPC-water	1:1000	Agilent
	glycerin jelly	undiluted	Ted Paella
	Histochoice	undiluted	VWR
	NBT/BCIP	1:50 in 100 mM Tris/HCl pH9.5, 100 mMNaCl	Roche/VWR
	NTMT	100 mM Tris/HCl pH9.5, 50 mM MgCl_2_, 100 mM NaCl, 0.1% Tween20	VWR
	phosphate-buffered saline (PBS)	1×	Gibco
	prehybidization solution	50% formamide 5× SSC (0.75 M sodium chloride/ 0.075 M sodium citrate dehydrate), 1× Denhardt’s (0.02% (*w*/*v*) each ficoll, polyvinylpyrrolidone, BSA), 0.1% (*v*/*v*) Tween 20 (Sigma), 0.1 mg/mL tRNA (Roche), 0.05 mg/mL Heparin	Amresco VWR VWR VWR Sigma Roche Alfa Aesar
	Shandon Immuno Mount^TM^	undiluted	TFS
	solution I	50% (*v*/*v*) formamide, 5× SSC (see above) 1% (*v*/*v*) sodium dodecylsulphate (SDS)	Amresco VWR VWR
	solution II	50% (*v*/*v*) formamide 2× SSC 0.2% (*v*/*v*) SDS	Amresco
	SuperBlock^TM^ (PBS)	undiluted	PBS
	TNT	0.5 M NaCl 0.01 M Tris/HCl pH 7.5 0.1% (*v*/*v*) Tween 20	VWR VWR Sigma
	True Black^TM^ (DMF)	1:20 in 70% Ethanol	Biotium
Probe generation	in vitro transcription	UTP-DIG labeling Mix	Roche #11277073910
		UTP-FITC labeling mix	Roche #11685619910
		T7-RNA polymerase 40 U/reaction	Promega
		RNA Protector 40 U/reaction	Roche
	template PCR	GoTaq Flexi 0.5 U/reaction	Promega
		dNTP mix	Promega

## Data Availability

The original contributions presented in this study are included in the article. Further inquiries can be directed to the corresponding authors.

## Supplementary Materials

The following supporting information can be downloaded at: https://www.mdpi.com/article/10.3390/ijms26209952/s1.

## Abbreviations

The following abbreviations are used in this manuscript:

*JTB*	*Jumping Translocation Breakpoint*
EDC	Epidermal Differentiation Complex
CPP/CPC	Chromosomal Passenger Proteins/Complex
*PAR*	*Prostate Androgen-Regulated*
AR	Androgen Receptor
AP	Alkaline Phosphatase
DAPI	4′,6-diamidino-2-phenylindole
UTP	Uridine-Triphosphate Nucleotide
DIG	Digoxigenin
FITC	Fluorescein Isothiocyanate
LOR	Loricrin
*SEDC*	Single Coding Exon EDC
*CREB3L4*	cAMP-responsive Element-Binding Protein 3-Like Protein 4
LAR	Luminal Androgen Receptor subtype
TNBC	Triple-Negative Breast Cancer
OASIS	Old Astrocyte Specifically Induced Substance
RISH	RNA In Situ Hybridization
IACUC	Institutional Animal Care and Use Committee
NACLAR	National Advisory Committee for Laboratory Animal Research
PBS	Phosphate-Buffered Saline
PFA	Paraformaldehyde
NBT/BCIP	Nitro Blue Tetrazolium/5-bromo-4-chloro-3-indolyl phosphate substrate
TB	TrueBlack^TM^
TFS	Thermo Fisher Scientific
