# Supplementary material for: Jumping Translocation Breakpoint Expression in Midgestation Mouse Embryos
Source: Int J Mol Sci. 2025 Oct 13;26(20):9952. doi: 10.3390/ijms26209952 (PMC12562924; doi:10.3390/ijms26209952)
Supplement: Supplementary file 1 [file ijms-26-09952-s001.zip › ijms-3889970-supplementary.pdf]

Supplementary Material

Probe sequences based on NCBI nucleotide data and BLAST results.

A. *Jtb* related

NM_206924.2 *Mus musculus* jumping translocation breakpoint (*Jtb*), mRNA

Exon 1 contains the translation start
GAGATGCAGTTGCCACCGAGTCCGGAACGACAACAGCAGCAAACAGACGAGAGCACCCGGGA
AGAGTCGGCGTCGCCAGTGCCGGGACCAGGAGCCTCCGCGAGCAAGATGGCCGCTCCCGAGAC
CGAGGCCGAGGGGCACGGGCACTAGGTCCGGACCCGCGTACCCCTTTCCCGGACGCCTGCGTCC
CCACAAAGACCTCGTGCTTCTAGCAAGACACCCGCATGGATTATTCTCGCTGTGGTCACGAGGCC
GTTCCTATAGCGCTCTAGTTGGCCCAGACTGAGAACTCTGGCATCTGGCTGCTCGGAGCTGGAAC
CTCAGGGAGGTCTGCTGCAACAGGTGCGACCCTGACCGCTCC**ATG**CTCGCGGGCGCGGGGAGGC
GTGGCCTCCCCCGGGCCGGCCACCTCTGTTGGCTGCTGTGCGCTTTCACCTTAAAACTCTG

Exon 2
CGAGGCAGAGGCTCCGGTGCGCGAGGAGAAGCTATCAG

Exon 3
TGAGCACTTCAACTTCGCCATGTTGGTTGGCAGAAGAGTTTGTGGTGACTGAAGAGTGTACTCCG
TGTTCTAACTTCCAGATT

Exon 4
AAAACAACACCTGAGTGTGGTTCTACAGGGTATGTGGAAAAAATCACATGCAGCTCATCTAAGA
GGAATGAATTCAAAAG

The breakpoint is located in the intron between exon 4 and 5.

Exon 5 contains the translation stop
CTGCCGTTCGGCTCTACTGGAACAACACTTATTCTGGAAATTTGAAGGCGTTGTGGTGGCTGTAGC
TTTAGTCTTCGCCTGCCTTGTCATCGTTCGTCAGCGACAACTGGACAGAAAGGCTCTTGAAAAAG
TCAGGAAGCAAATTGAGTCCATATAGC**TGA**ATTTCTACTGTATTATGGTGGCTCTTTACGGACTGT
ATCTCAGATGGAGAAAGTTCAGCGGCTAATTTGCACTCGTAAACCTGTGGTAGTAGCATTTTCAA
TGACTTCCTCTTCTAGACCATTAATGAGCACAATAAAAAGTAAAATAGAGCTGGACATGACACA
GACTTGGAATCCCAGCACTTGAGAGAATGAGGCAGGAGGATCCTTGGTAACTTTGTAGGCCGAC
TAGGCTGAACTACATAGTAAGCCTGTCTTTAAAGAGAAAACGGAAATAATAAAGGAGTAAAGA
GTTTC

Probes:

m*JtbA* RISH probe sequence is located in the 5′UTR upstream of the breakpoint

AGCCTCCGCGAGCAAGATGGCCGCTCCCGAGACCGAGGCCGAGGGGCACGGGCACTAGGTCCG
GACCCGCGTACCCCTTTCCCGGACGCCTGCGTCCCCACAAAGACCTCGTGCTTCTAGCAAGACAC
CCGCATGGATTATTCTCGCTGTGGTCACGAGGCCGTTCCTATAGCGC

m*JtbB* RISH probe sequence is located in the 5′UTR upstream of the breakpoint
GACGCCTGCGTCCCCACAAAGACCTCGTGCTTCTAGCAAGACACCCGCATGGATTATTCTCGCTG
TGGTCACGAGGCCGTTCCTATAGCGCTCTAGTTGGCCCAGACTGAGAACTCTGGCATCTGGCTGC
T

m*JtbE* RISH probe sequence is located in the last exon and 3′UTR downstream of the breakpoint including the translation stop.
TGCCGTTCGGCTCTACTGGAACAACACTTATTCTGGAAATTTGAAGGCGTTGTGGTGGCTGTAGCT
TTAGTCTTCGCCTGCCTTGTCATCGTTCGTCAGCGACAACTGGACAGAAAGGCTCTTGAAAAAGT
CAGGAAGCAAATTGAGTCCATATAGC**TGA**ATTTCTACTGTATTATGGTGGCTCTTTACGGACTGT
ATCTCAGATGGAGAAAGTTCAGCGGC

B. *S100a1* related

AF368423.1 *Mus musculus* S100 calcium binding protein A1 (*S100a1*) gene, complete coding sequence
GATCTAGAAAGGGACTCTTCAAACTCCTCACATCCACAACCCACAAAGTGTTTGGCCTGGACAC
CAGGGCCTTCGGGGGTCCACGAAGGGAAGAAACATGCCCACTCGTCCCCTCATTTCTGTCTGTCC
ACCCACCCACATACTCCTCCGGGGACCGTTGCCTGCTCTCCTAAAGTCACTTCCTGAATCTCTGCC
TGAACCCGCTTGGAAGGAGGGACCCGGGAGCTGATAAAGAAAGGAAGGTCTCCAGGTTCTCTGC
AGTCAGGAAAAGTAACTCACCGGGAAAGAAGGTGGGATGCAGCTAACCGATCTGGAGCCCTGA
GGCTGAAGTTTATAAGCACCAGGCAAGGCGAGAGCCACTCCCAAGTCTGAAGTGTCAAGTTTCC
GCCTCCGCTGAGGGGACCGCCCCTGCTGTCACTAGCCATCCCAACACAGCGCCACGCGACTGGA
CACAGCCTGATCAGGAAGGACCACAGGGTCTCATCCCTCCCAATTATGAGGGCTACTTGGGAGT
GTGTTAGAGGGACTGCTACTGCTGCTTGAAAAAGATTAAAAAGAAAAGCAAACAAAATATACTT
GGGGGATTCCAGCACTGAAAAATCCCCTTCTGAGAAGGCAAAGAGATCCAAAGGCACTCGGAG
AGCCCCAGACTAAGGGTCCCATAGTGGAACTCCTATTGAAATTCTTCACTGAGTTCCGAGACAGA
AGCCCCTGTCGGAGGCGCACAGGCTAAGCACTCCTGGGCAGAATCCTTCCCTTTCCCTGCTCAGC
CCGCACCGCAGCACCCAGCACCATAGCACCCTTCCCTCGACCTCCTTCCCTTCCACCTACTTCCCG
CTAGGCTCAGGGAGGTGGTGCAAGCAAGAACGGAAAGAAACACAGGACAGCCCCTTCCCTCCT
GGGAGACAGAGGGCGCCTCTGTCCACTCCTGGCCCTTAGCCCTTTGGGTGTCGTTTGTGAAGGGG
TGGAGCCGTTGGGGAGGAGGTCGGTAGGGAAAGACGACTAAAAGACAGGTCTCCTCACACCGG
TCCAGTGGCCACATTTGCAGCGTGCCCTTCTGTCGAGAATCTGTTCCGACGTCAGGCCGAGGCCA
ACCGTGAGTATCCTACCCTGCTTAGGATAGGTCTCTCTGGCTTAGCCAGCCTGGAAGAAAGGGAA
CTTCAGAAGGACCCCAAGAGCCCTGGAAAAGGATACTGTGGTGTCCGGGAGGAGTCGGTCAAG
GGAAAGAGCAGTTCGGGAACTAGGGATGATGAAGTTTGGAAAGTAGTGAGAGGAGGTGAGTGA
AGGTCAGAGAGCTGGGAGCGTGTGGTGAATGAGGGCGCCACCCAGCTTCCAGGCTACCAGGTCT
TCCTACACAGAATCCTGTGTCTCCTGCCAGGGACGCCAGTCTCTAGGTGTGGGCAGGGAGGAGAT
ACAGCCCTGCTCCTTCAGGGTAAGAATAAGAAAAGCTGTCCCTGCCCCTTCTCCACCCTCACTCT
CTGCCTTCTCAAGAGCCAGGGAAGGCTCCTAGCAGTGGAGCTTAACGTCATGTGGTATCTTTCTT
AAAGAAGGGGGTCTGTGGCGGTGTGCACTGTCCCATCTCTGCCTCACAGACCTCCTCTCTAGCC
CTTCTTTCTCAGCCTTGACCCCCTCTCCTCTCTCCCCTAATTTTTCCCTCTTTGTTTCCCACTGCTCC
AAGCCCAGATCCTCCCCTACATAAAATTCCAAGCTGCTAGAGGGTTCCAGGCAACCTCCAGATG
CCCCTCATAGATGAGAATGGTAACCGAGCCGCCCAAATTCCTCCCTTGAGCACCCTCTTATCCCT

TTGATCAGCCTGGCTTGAATTCTCTCTCCCACTGTCTCTGTAACAACTGCTTCTTGTGACTGGAGTT
CAGGTGACAGTCACAAGGGCTCCACTGAAGAGCTGGCATGTCATCTAATCAGGACCCATTGTCC
AGAATACTCAGTGGTGAAGAAGGATGAGGACACCTGAGCTGAGACACATGAGCAACCCCTGAG
GCTGTGGAAGGCATGACCCTCCCAGGGCCGAAAGATCGAGCCCCCCACCCCACCCCAGTTTAGT
AGTCTCTTCTCAATAGACTTCTCCATTCTCCCACTCTCGCCCAGCCCTTGAGCTCAAATGACCTCA
TACATTCAGCCCTTTATCAAACACAAACCAGCCCTAGCCTGCTCCCACTGGAGCTACCGAACCAA
CGCTGGCCCGTGGCAGCAATGGACAGCATCAGGAAGAACACCTACAAAGCTCGTCTCCAAGGA
AAGCCTCAGACATCTCTTCTCCCTTCCTTCTACCTACCTGATGGAAAGGGATGATTTTGCAGAGAA
AAGGACTAGAACAGGCAGTCATGTTAAGAATACCGCCATACCGCCCCCCCCACCCCCGTCCCCC
AAAGAAGCCAAAATGGCATGCTGAAGAGAGAGGGGGAACTCAGACCAGTAATCTCAGCACACA
GAGACACGACTGCCCAGAAGCTAAGGATCTGCTTGAGCTAATTCCAGGACAGCACAGGCTACAG
AGTGTGACCCTGCTATAAATAAATAAATCCCAAGCACAAAAAGATGGAGATGTAGCCAAAGGG
ACCTGGGCTGAATCCTCGGTATCTTAAGAGGAAGAGCCATGCTTGCTGACAGGAAGCTTGGGAA
CAGTAAGACACCTCTTCCTCACTGACCCCCCATCAGCTCACCAAAGGTATCAGACTTCTTCCTGG
GGTAGCAGAGGAGATCGTAGCCCACTTAGTAGCAACAACAAAGCTTAACTGTGACAGGCCTGGA
AGTAAAACTAGAGCTCGAAGCCTGCCTGGTCCTTGTGTCCACCCATGTCTCCCCTCCCGCTCCCC
AGCCTTGAGTCAGCCACTGCAGATGTCTTTTGCAGGCTTCTGAAGACAGTCCAGTTCCTATTGCTT
CATCTCCATGCCTATCCCTGACTCCCTCTGACGGGGAAATGATAGAGGGACCCAAAACGCTGGA
CCAGATTTAGTGCTAGGGTTTCTCCTACACTGCAAAATCTGAATGAAAGATAAAGTACAATGAGG
TTCATCTCGCAGCTATTTGAGCGAATCCAGGGCCAGGGCTGAAGATTTCCCTTACCCAGACCACA
GATGTCCTGAATCTGGTTTATAATTCACTTCCATCTTTGTGGGAATGGGTAGGTGTGCTGCTGAA**A**
TGGGCTCTGAGCTGGAGAGTGCCATGGAGACCCTCATCAATGTGTTCCATGCCCATTCGGGCAAG
GAAGGGGACAAATATAAGCTGAGCAAGAAAGAACTGAAAGACCTGCTACAAACTGAACTTT
CTGGCTTCCTGGATGTAAGCACAGGATTGAGGTACAGGGTGGAGGAAGGGGATGTAGCCCCAGC
TGGCATGGAACTTGCTATGTAGATCAGGCTGGCCTTGAACTCAAATAGACCTACCTGCTTCTCTCC
CCCACTGAGTGCTAGGATTAAAGGCATCGCTGTGCCCTAAAATGATTTTGTGTGTGAATTTTTAAT
TAAATGAAAGAAAGTCTGTAAGGTTCCCAGCTTGTCACAATGTAGACAATCATGTATGACAACA
GATGTAACTATTAACTCTGCTTAGTTCTATAGGTCACCAGTTTTTGGTTTTTTTTTTTTAAGATTTAT
TTATTTATTACATGTAAGTACACTGTAGCTGTCTTTAGACACATCAGAAAAAGGCGTCAGATCTTG
TTACGGATGGTTGTGAGCCACCATGTGGTTGCTGGGATTTGAACTCCGGACCTTTGGAAGAGCAG
TCGGGTACTCTTACCCACTGAGCCATCTCACCAGTCCCAGGTCACCAGTTCTTAACTCACCAGTCT
TTAAGGTTGTAGAAGACTCCCATAATTAAATATCATTCTCTCACTTGCTCATTTCCACCTCCTCCTC
CACCACCATGGTGGCTCTGACTTAGTGATAAACACTTCCATCCCCTTCTACAGGTCCAGAAGGAT
GCAGATGCTGTGGACAAGGTAATGAAGGAACTGGATGAAAACGGAGATGGGGAAGTGGACTTC
AAGGAGTATGTTGTGCTGGTGGCTGCTCTCACAGTGGCTTGTAACAACTTCTTCTGGGAGACCAG
T**TGA**GCACAGTCTGTGAGCAGTGCCCTTCCTCTCCCACTTGTCAAGATTTCCCCTTTAGCGTGCTT
GCTCCTCACCCCACCTGTACCTCCCTATGTCCCCATCCTTGCCCAGTCTCGACGTGCACCAAGGGC
ACAAGAGTGGCAGTCCAGATCTGCCACTTGTTTTAATAAAGCCTTCTCTCTCACCAGCTATCAAT
GTCTGTACCCTCTGGGCATGGCTATGTGTGAAGTAAGGGGTGCTCACTCCCAACACCTTATCTTGT
CAAGTTTACATTAGTTCCTTATTTAAGGAACACTCAAAACCCAGAAATGTAGGGGTCCGAAAATT
TTAGCTGGAATCCAAGTGTCATTGGCTTCTCAAGGATTTGTATGAGTAATTGGTAATACCAGGACT
TGAAGGATGAGGCAGGAAGATTTTGAGTTCCAAGCCAGCCCATACTACATAGCCAGAAACAGA
AATAAACAAGCCATCAAAAAATGTGGGGCTGGTGAGATGGCTGAGTGGGTAAGAGTACCTGACT
GCTCTTCCAAAGGTCCGGAGTTCAAATCCCAGCAACCACATGGTGGCTCACAACCATCCGTAAC
GAGATCTGGTGCCCTCTTCTAGTGTGTCTGAAGACATCTACAGTGTACTTACATATAATAAATAA
ATAAATCTTTTTTAAAAAATCTGAAGAAAGCTAGAAATGGTGGCACGTACCATAGTTAACACCA
GCTCAGCACCTGGGACGTAAAAGGCAGGAGGATCTCTGTGAGTCCAAGCCCAGCCTGGTCTGTA
TAGAGAATTCTAGAACAGCCAGAACTACATTATAGAGACTCTGTCTCAAAAAAACTACCAGAGG

CAGTGGTGACACACGCCTTTAATCCCAGCACTCGGGAGGCAGAGGCAGGCAGATTTCTGAGTTC
AAGGCCAGCCTGGTCAACAGAGTGAGTTCCAGGACATCCAAGGCTACACAGAGAAACCCTGTCT
CGGAAAAAAAAAAAAAAAAA

Probe:

m*S100a1* RISH probe sequence is located in the last exon containing the translation stop and part of the 3′UTR.
TATGTTGTGCTGGTGGCTGCTCTCACAGTGGCTTGTAACAACTTCTTCTGGGAGACCAGT**TGA**GCA
CAGTCTGTGAGCAGTGCCCTTCCTCTCCCACTTGTCAAGATTTCCCCTTTAGCGTGCTTGCTCCTCA
CCCCACCTGTACCTCCCTATGTCCCCATCCTTGCCCAGTCTCGACGTGCACCAAGG

C. *Sox10* related

NM_011437.1 *Mus musculus* SRY (sex determining region Y)-box 10 (*Sox10*), mRNA
AGTCAGTCTCGGCTGTCCAGCCAGGGTGTTTGGTGGTGAGGATTCAGGCTCCGTCCAGACAAGGC
AGTGGCCTGAGGCTCAGGGCCCCCCAGCCCCTCCCTCCCAGTCCATCAGCGTCACTCCCCAGCCC
CGAGCTGGACCGCACACCTTGGGACACGGTTTTCCACTTCCTCAGGACGAGCCCCAGACTGGAG
GAGAGGTCGGAGGAGGTGGGCGTTGGGCTCTTCACGAGGACCCCGGCGGCGGGCCCGGGGGAG
GCGGCCGAAGCGGCGGCGGCCGGGAGCGAC**ATG**GCCGAGGAACAAGACCTATCAGAGGTGGA
GCTGAGCCCTGTGGGCTCGGAGGAACCCCGCTGCCTGTCCCCAGGCAGCGCGCCGTCGCTGGGA
CCCGACGGCGGCGGCGGTGGCTCGGGCTTGCGAGCCAGCCCGGGGCCCGGTGAACTGGGCAAG
GTCAAGAAGGAACAGCAGGACGGCGAGGCGGACGATGACAAGTTCCCCGTGTGCATCCGCGAG
GCGGTCAGCCAGGTGCTCAGCGGCTACGACTGGACGCTGGTGCCCATGCCCGTGCGCGTCAACG
GTGCCAGCAAGAGCAAGCCGCACGTCAAGAGGCCCATGAACGCCTTCATGGTGTGGGCACAGG
CGGCACGCAGAAAGCTAGCCGACCAGTACCCTCACCTCCACAATGCTGAGCTCAGCAAGACACT
AGGCAAGCTCTGGAGGTTGCTGAACGAAAGTGACAAGCGCCCCTTCATTGAGGAGGCTGAGAGG
CTCCGGATGCAGCACAAAAAGGACCATCCGGACTACAAGTACCAACCTCGGCGGCGGAAGAAC
GGGAAGGCAGCCCAGGGGGAGGCAGAATGCCCAGGCGGGGAAGCCGAGCAAGGAGGGGCTGC
TGCTATTCAGGCTCACTACAAGAGTGCCCACCTGGACCACCGGCACCCAGAAGAAGGCTCCCCC
ATGTCAGATGGGAACCCAGAGCACCCCTCAGGCCAGAGCCATGGCCCCCCAACCCCTCCAACCA
CCCCAAAGACAGAGCTGCAGTCCGGCAAGGCAGACCCCAAAAGGGATGGGCGCTCCTTGGGGG
AGGGCGGGAAGCCCCACATCGACTTCGGCAACGTGGACATCGGGGAGATCAGCCACGAGGTAA
TGTCCAACATGGAAACCTTTGATGTGACTGAGCTGGACCAATACCTGCCACCCAACGGGCACCC
AGGCCATGTGGGTAGCTACTCGGCAGCTGGCTACGGGCTGGGCAGTGCCCTGGCTGTGGCCAGT
GGACACTCTGCCTGGATCTCCAAGCCACCAGGTGTGGCTCTGCCCACGGTCTCGCCCCCTGGTGT
GGATGCCAAAGCCCAGGTGAAGACAGAGACCACAGGCCCCCAGGGACCCCCACACTACACCGA
CCAGCCGTCCACTTCCCAGATCGCCTACACTTCCCTCAGTCTGCCCCACTACGGCTCCGCCTTCCC
CTCCATCTCACGACCCCAGTTTGACTATTCTGACCATCAGCCCTCAGGACCCTATTATGGCCATGC
AGGCCAGGCCTCTGGCCTCTATTCAGCCTTTTCTTACATGGGGCCCTCCCAGCGGCCCCTCTACAC
TGCCATCTCTGACCCCAGCCCCTCAGGGCCCCAATCCCACAGTCCCACACACTGGGAGCAGCCA
GTATATACGACTCTATCCCGACCT**TAG**AGGGGCCCTGTCACCACCAGTGCCCACAGGGCCTTGGG
GCCCCTTCCCCCAGCCTGTGTGCCCTGCTCCTCATCAGCCTCAGGCTTGGCAGTGGAGGAGGCTG
CAGAGGCTGACAGCAGCAGGAAGGCTTCTGCCAGCTCCTGCCCTGATGACCTCCACCCACCCCG
GCTCTGGAGGCCAAGCCCTGACTGAGCTGGCAAAGGAAGTTTGGTGGCTCTCCCAGACTGAGGG
TGAGGGGCCACCCTTCCCCAGAAATGACCCTCTATCCCAGGACCTGAGAGTCCTGCTCAGTCCTT
CAGGGAGGCAGGAAGGGTTAGGGTAGGAGGCCTCACTGCTCCTGTTATCTCCTTGGAGAGCAAG

GGGCCCGTGTGCTACTGTGTCATTGCCTAAGAGCTAAGCTGCCCAGGGACCTGCCTCAAAGCCTG
GAGCTGGTTCTGTCCTGTCACTAAGGACGCACTGAGGACAGCTTTGAACAGCTTTGTAAAGAGCA
GGGTGGGACACTCTGATCCTTTCTCCGCCCCCTCTTCACCTGAATGTGGGAACTGGTCGAGGTCCC
CAGATCCAGTTCCGTGTCAATAACCTCATCCCTTGCCTAACTGAGGAGAGTCCCCATGTTCTTCCC
ATCCCACCCCAGGTGAGGAGCCAGGGGCTCCAGGAAAGAGTCAGAGGCAATCCACCTAGCCTTC
TTCTCGCCTTCTGTCAGCCTCAAGCCCCCAGGCTCAGAGTTAGCATGGCACGGCCCTAGCCCCTG
GGTTGCTGGCAGCAGGCTGGACACTAAACCCCTGCCATGTACAGTCTACCTTTGCCTTGCACCCTT
TGCTCCAGCGATACCTTAATAAAGTACGTAGCTCTGTCTCCCCATCTCCTGTCTGCAGCCCAGTCC
ACATGTAACACATCGCTGCCCCTTTATTTATCTCCATAGTCGCCATGTTCCTATCCCTCTAGCTACT
CCAGCCCCGATTAACTTGCATTAAGCACTCCAAGTAATAAAATCAATGCTTCTGGTTGCCTGCTT
GGTGGCCTGA

Probe:

***mSox10* RISH probe sequence is located in the 5'UTR**
TCCAGCCAGGGTGTTTGGTGGTGAGGATTCAGGCTCCGTCCAGACAAGGCAGTGGCCTGAGGCTC
AGGGCCCCCCAGCCCCTCCCTCCCAGTCCATCAGCGTCACTCCCCAGCCCCGAGCTGGACCGCA
CACCTTGGGACACGGTTTTCCACTTCCTCAGGACGAGCCCCAGACTGGAGGAGAGGTCGGAGGA
GGTGGGCGTTGGGCTCTTCACGAG

D. Foxd3 related

AF067421.2 *Mus musculus* HNF3/forkhead homolog 2 (*Foxd3*) mRNA, complete coding sequence

GAACTCAATCGAGGAAGGCTGGTGAAGTGAGGGGGTGCGGCGTGGAGAAACCTCCGGGGTCGC
GAGCAGTAGCTGTCTCCTCGTAGAGAAGCGTCGAGGACCGCAAGAGTTCGCGGACGCGGGCTGC
GGACGCAGCCCCACACCGCTGCCAGGCCTCCTCGTGTGAGCTGACCCGACAGCCCCGAGCTACC
TGTGCCCTCTCCCCCCCTCCCCCGGCTTTCTTTCGGGGGACACTCACACCTCCTTTGCCTGAGCTCC
GCGCCGCCCCTCGCGGCCCCCCTCGCTCTCTGCTCGCTCCTGGCCGCCGCCACCAACCCCGCGGA
GGG**ATG**ACCCTCTCCGGCAGCGGCAGCGCCAGCGATATGTCCGGCCAGACGGTGCTGACGGCCG
AGGACGTGGACATCGATGTGGTGGGCGAGGGCGACGACGGGCTGGAGGAGAAGGACAGCGAC
GCCGGGTGCGACAGCCCCGCGGGGCCTCCGGACCTGCGCCTGGACGAGGCGGACGAAGGGCCA
CCGGTGAGCGCTCATCACGGCCAGTCTCAACCGCAAGCCCTGGCACTGCCCACCGAGGCGACCG
GGCCGGGGAACGACACGGGAGCTCCGGAGGCCGACGGCTGCAAGGGCGGCGAGGACGCGGTG
ACAGGCGGCGGTGGGCCCGGCGCGGGCAGCGGGGCGACGGGAGGTCTGACCCCGAACAAGCCC
AAGAACAGCCTGGTGAAGCCACCCTACTCTTACATCGCGCTCATCACCATGGCCATCCTGCAGA
GCCCGCAGAAGAAGCTGACCCTGAGCGGCATCTGCGAGTTCATCAGCAACCGTTTTCCGTACTAC
CGGGAGAAGTTCCCCGCCTGGCAGAACAGCATCCGCCACAACCTGTCGCTCAACGACTGCTTCG
TCAAGATCCCCCGCGAGCCGGGCAACCCGGGCAAGGGCAACTACTGGACCCTGGACCCCCAGTC
CGAGGACATGTTCGACAACGGCAGCTTCCTGCGGCGCCGGAAGCGCTTCAAGCGCCACCAGCAG
GAGCACCTGCGCGAGCAGACGGCGCTGATGATGCAGAGCTTCGGAGCCTACAGCCTGGCGGCGG
CGGCGGGCGCAGGACCCTACGGCCTGCACCCCGCGGCGGCTGCGGGCGCCTACTCGCACCCCGC
GGCGGCGGCGGCGGCCGCGGCTGCTGCGGCGCTCCAGTACCCGTACGCGCTACCACCCGTGGCT
CCCGTGCTGCCGCCCGCAGTGCCCCTGCTGCCCTCGGGCGAGCTGGGCCGCAAAGCGGCCGCCTT
CGGCTCGCAGCTCGGTCCGAGCCTGCAGCTACAGCTCAACACCCTGGGCGCCGCCGCGGCAGCC
GCGGGCACGGCGGGCGCGGCGGGCACCACGTCGCTCATCAAGTCCGAGCCCAGTGCGCGGCCGT

CGTTCAGCATCGAGAACATCATAGGCGCGGATCCGCGGCGCCCGGGGGCTCGGCTGGGGGTGG
TGGGAGCGGCGGCGGAGCCGGGGGTGGCGGGGGCAGTGGGGGCGGCGGCGGCGCGCAGTCCTT
CCTGCGGCCGCCCGGGACTGTGCAGTCGGCTGCGCTCATGGCCACGCACCAGCCCCTGTCGCTGA
GCCGGACGACTGCCACCATCGCGCCCATTCTGAGCGTGCCGCTGTCTGGACAGTTCCTGCAGCCC
GCAGCCTCAGCCGCAGCGGCTGCGGCCGCCGCCGTGCAAGCCAAGTGGCCCGCTCAA**TAG**TGAT
GAGCTAGTGGCCGGGCCGGACGGCGGCCTCTGGCAGCAGCCTGCTCTTCCAGGCGGTACGCCCT
CTCTGATCCTGGTCCATCTGTCCTGGTCCTGGTCCTGGTCCCTCTACCCAATCCTGGACTCTGCTAC
CCGCCCCCGACTCCCCAACACTGACCAACAGCGGCCTCCATCCCCTCACTCACCTAAGCTGGCG
GAGCAAACTCAACCCGTCCGCTGGGAATAACTTTCCAGTACAGGTAAAACTGATAACCGAATTT
CCAAAAATGCACCCCGACGGCGCCGTTGTTCTCAGTACTGTGGGGGTGGGAGGGACATTCTTTGT
ACATATTTGTATAAAAATCATTGACTTTCCTTTTGGGGTTTTTATTTTTTTAAGAAAATAAAAAGCA
ATTCAGTAGATTTAGAGCTTTGAACTTTCATATTTTTTTGAAGGTTCACTCTGCGCAGTTTTATCTG
AGAAAAGAATGTATAAAGACTTGGGAGATTTAAATACAAAAAATTTTTCAAAAAGGCGAAGTGT
CATTGTATTATAAAAGTCTGTTTATATATGAATGAATATATATGGTATTCTAAATGAGCGGC

Probe:

***mFoxd3* RISH probe sequence is located in the 3′UTR**
AACTCAACCCGTCCGCTGGGAATAACTTTCCAGTACAGGTAAAACTGATAACCGAATTTCCAAA
AATGCACCCCGACGGCGCCGTTGTTCTCAGTACTGTGGGGGTGGGAGGGACATTCTTTGTACATA
TTTGTATAAAAATCATTGACTTTCCTTTTGGGGTTTTTATTTTTTTAAGAAAATAAAAAGCAATTCA
GTAGATTTAGAGCTTTGAACTTTCATATTTTTTTGAAGGTTCACTCTGCGCAGTTTTAT

E. *Rab13* related

NM_026677.4 *Mus musculus RAB13*, member RAS oncogene family (*Rab13*), transcript variant 1, mRNA
GAGGGAGGAAAACTTCCTGGCTGGGACTCCGGCGGTTTCTGCCTGCCAACCCTCGGATCCCGCCT
ACCAGTGTTGGCTCTTCCCGACCCCTCCCCCGGCGCCCCCAGTGTCCGCC**ATG**GCCAAAGCCTAC
GACCACCTCTTCAAGTTGCTGCTCATCGGGGACTCGGGGGTGGGCAAGACTTGTCTGATCATTCG
CTTTGCAGAGGACAACTTCAACAGCACTTACATCTCTACCATCGGAATTGATTTCAAGATCCGAA
CCGTGGACATAGAGGGGAAGAGGATCAAACTGCAAGTGTGGGACACGGCTGGCCAAGAACGAT
TCAAGACAATAACTACCGCCTATTACCGTGGAGCCATGGGCATTATCCTCGTATATGACATCACA
GATGAGAAATCCTTCGAGAATATTCAGAACTGGATGAAAAGCATCAAAGAGAATGCCTCTGCGG
GAGTGGAGCGCCTCCTGCTGGGAAACAAGTGTGACATGGAGGCCAAGCGGCAGGTGCAGAGAG
AGCAGGCGGAGAAGTTGGCTCGAGAGCACAGAATCCGATTTTTTGAGACGAGTGCCAAATCCAG
TGTGAATGTGGATGAGGCTTTCAGTTCCCTGGCCCGTGACATCTTGCTCAAGACAGGAGGCCGGA
GATCGGGAACCAACAGTAAGCCCTCAAGCACTGGCCTGAAAACATCTGACAAGAAGAAGAACA
AGTGCTTGTTAGGC**TGA**GAGCATTTCTTGCCTCCTATTCACCCTGAACCTGGAGGCTAGACCTGA
GGGAGTCGGACTGAGGGATTGCAGATGGGAGAACTGTGGTGGCACCTCAAGGGGAGATGAGGG
GAATAAGGAGACCGGCGAGGACGAGACGGAAGAAAGGGGCAGGGAAAGGAGGGGGAGGAAC
CAAGGATGTGAAAGGTGAACAGAAGGGATTTGAGAAGAGGAAAGGAAGAAGAAATGAATGGC
TCAGGCCTTGGACAGTCCAACATTAAAGTCAACATGCTGATCTCTCCATTCCTGGTTCAGGGTTA
GGGTCCTGAGAGGCTGGCTCGGCACTACTCCGAGGGTCCCTCACTCTACAAGGTCTTTGTTAGTA
TTAAAGGCCACTGTTTTGCATGAATGTCCCATTTGCATTACTTTCATTATTGTCAGAATTGCTCTTC
ACTCAAATCCTATTTTTGTCACGCCAAGATATTGGTTCACCTGAATGTGGCTGGGTTCCCCTTCCTT
GCCCCAACTCTTTCACTGGTGATGAAAACAGCATGGGGCAGCCTGAAGGACGGACATCCTGTTTC

CACTGTGGGTTCCCAAGGACTACAAGAGTGGACGGAACCTTGCCTTGAGCACACAGTAACCCAA
GGACAAAGGATTTGAACCAGGCTTCAGTAAACAGCAGCACTTAGTATGGTTTATCCAAGGAGAT
GTGGGACATCTTTGATTCTGATGTAGTCAGCTTAGGTGTTGGGTACTGTTAGCTGCTTTTGTTAGA
GTATTCTCAGTGTTGCACAAAGAAATACATGAACAAGGTGAGAGTAAAAAAAAAAAAAAAAA
AAAA

Probe:

***mRab13* RISH probe sequence is located in the 3'UTR**
TGGCACCTCAAGGGGAGATGAGGGGAATAAGGAGACCGGCGAGGACGAGACGGAAGAAAGGG
GCAGGGAAAGGAGGGGGAGGAACCAAGGATGTGAAAGGTGAACAGAAGGGATTTGAGAAGAG
GAAAGGAAGAAGAAATGAATGGCTCAGGCCTTGGACAGTCCAACATTAAAGTCAACATGCTGA
TCTCTCCATTCCTGGTTCAGGGTTAGGGTCCTGAGAGGCTGGCTCGGCACTACTCCGAGGGTCCCT
CACTCTACAAGGTCTTTGTTAGTATTAAAGGCCACTGTTTTGCATGAATGTCCCATTTGCATTACTT
TCATTATTGTCAGAATTGCTCTTCACTCAAATCCTATTTTTGTCACGCCAAGATATTGGTTCACCTG
AATGTGGCTGGGTTCCCCTTCCTTGCCCCAACTCTTTCACTGGTGATGAAAACAGCATGGGGCAG
CCTGAAGGACGGACATCCTGTTTCCACTGTGGGTTCCCAAGGACTACAAGAGTGGACGGAACCT
TGCC

E. **BLAST results** for above probes based on a NCBI search on 20250930 with a cut-off at 90% similarity.

Probe	Target description	Accession numbers	Identity
JtbA	*Jtb* related	AK146337.1, BC008139.1, AK089048.1, NM_206924.2, AK004469.1, NM_206924.2, AK004469.1	100%
	Mus musculus chromosome 3	AC096623.31, AC096622.14, AC119825.11, NM_206924.2, AK004469.1	100%
	Mus musculus Rab13 targeting related	JN946420.1, JN962204.1, N962204.1	100%
	Jtb related in other *Mus sp.* or other organisms	AB016490.1, XM_021158417.2, XM_031376054.1, XM_052179944.1, XM_034499352.2	90-99.99%
JtbB	*Jtb* related	AK146337.1, BC008139.1, AK089048.1	100%
	Mus musculus chromosome 3	AC096623.31, AK184177.1, AC096622.14, AC119825.11	100%
	Mus musculus cDNA, Clones Y0G0129A05, Y0G0145C07	AK184177.1, AK188418.1	100%
	Mus musculus Rab13 targeting related	JN946420.1, JN962204.1	99-100%
	Jtb related in other *Mus sp.* or other organisms	AB016490.1, XM_021158417.2. XM_021196596.2,	90-99.99%
JtbE	*Jtb* related	AB016490.1, BC008139.1, NM_206924.2, AK004469.1	100%
	Mus musculus *Slc39a1* targeting related	JN949480.1	100%
	Mus musculus chromosome 3	AC096623.31, AC119825.11	100%
	Mus musculus clone s: mgs1-182e11, Y2G0135J01	AC096622.14, AK215601.1	100%
	Mus musculus Rab13 targeting related	JN946420.1, N962204.1	100%

	Jtb related in other *Mus sp. o*r other organisms	AK146337.1, AK089048.1, XM_021158417.2, XM_021196596.2, XM_031376054.1, XM_028753235.1, XM_034499352.2	90-99.99%
S100a1	*S100a1* related	BC005590.1, AK012578.1, NM_011309.3, AF368423.1, AK168822.1, JN945419.1, AK002721.1	100%
	Mus musculus chromosome 3	AC160552.13	100%
	Mus musculus *2500003M10Rik* targeting related	JN953600.1, JN960058.1	100%
	S100a1 related in other *Mus sp. o*r other organisms	AK017279.1, XM_021157213.2, AF087687.1, XM_021196637.2	90-99.99%
Sox10	*Sox10* related	NM_011437.1, XM_034516963.2, AJ001029.1, AK042542.1, BC062067.1 NM_019193.3, AF017182.1, AK043220.1, NG_050695.1, JN959745.1, JN959313.1 BC025171.1,	100%
	Mus musculus chromosome 15	AL591921.6	100%
	Sox10 related in other *Mus sp. o*r other organisms	XM_060389064.1, U66141.1, XM_055602977.1, XM_021628556.2, XM_052161299.1, XM_029548204.1, XM_029470086.1, XM_028789912.1, XM_003510279.4, XM_027403983.2, XM_005066979.4, XM_051159956.1 XM_036209024.1, XM_028871123.2 XM_075959913.1, XM_005354207.3 and many more	90-99.99%
Foxd3	*Foxd3* related	AF067421.2	100%
	Mus musculus transcription factor Genesis	U41047.1	100%
	Mus musculus chromosome 4	BX005053.5	90-99.99%
	Foxd3 related in other *Mus sp. o*r other organisms	NM_010425.3, XM_021161526.1, XM_021200531.1, XM_031377923.1, XM_052175581.1, XM_034503650.2, NM_080774.1, XM_028778755.1, XM_051188221.1, XM_052717918.1, XM_036180172.1, XM_028870329.2, XM_005074328.4, XM_076564558.1, XM_075945306.1	90-99.99%
Rab13	*Rab13* related	JN946420.1, AK080805.1 NM_026677.4, NM_001293741.1 JN962204.1, BC027214.1	100%
	Mus musculus strain 129/SvJ clone mgs1-182e11	AC096622.14	100%
	Mus musculus chromosome 3	AC096623.31, AC119825.11	100%
	Rab13 related in other *Mus sp. o*r other organisms	XM_021158101.2, XM_021158102.1 AK002303.1, XM_021196250.2, XM_028753087.1, XM_028753085.1, XM_034499763.2, XM_031374595.1 XM_031374594.1	90-99.99%
